# Two-Level Regular Designs for Baseline Parameterization

**DOI:** 10.3390/e27070706

**Published:** 2025-06-30

**Authors:** Shengli Zhao, Mengru Qin

**Affiliations:** 1 School of Statistics and Data Science, Qufu Normal University, Qufu 273165, China; 2 Zaozhuang No.2 Middle School, Zaozhuang 277000, China

**Keywords:** baseline parameterization, K-aberration, two-level regular design, word length pattern

## Abstract

This paper considers two-level regular fractional factorial designs for baseline parameterization. Some new results that reveal relationships between the *K*-values and word length pattern are developed. The new results help find two-level regular fractional factorial designs that are likely to be optimal under the *K*-aberration criterion. Illustrative examples are included to demonstrate this point.

## 1. Introduction

The application of experimental design in information theory is primarily reflected in optimizing experimental schemes to maximize information acquisition efficiency while minimizing uncertainty. The core concepts of information theory, such as entropy, provide quantitative tools for experimental design. For example, in the field of communications, experimental design can optimize channel coding schemes, thereby improving transmission reliability. Additionally, in machine learning feature selection, experimental design based on information entropy can identify the most discriminative feature subsets, reducing model complexity and enhancing classification accuracy. The integration of experimental design and information theory enables a systematic approach to solving key challenges in information acquisition, processing, and optimization, offering theoretical guidance for engineering and scientific experiments. Bose explored the interplay between statistical experimental design and information-theoretic concepts, discussing how to construct designs that maximize informational yield from data [1]. His work established parallels between statistical efficiency (particularly variance minimization in parameter estimates) and information-theoretic principles.

Factorial designs have a wide application in various fields. The baseline parameterization (BP) model and the orthogonal parameterization (OP) model are two of the most used models in the analysis of experimental data. The BP model is a linear model based on baseline constraints while the OP model is based on zero-sum constraints. There are numerous research findings under the OP model. For details, please refer to [2,3] and the references therein. BP is quite a natural option for modeling the experiments with each factor having a null state or baseline level. For example, in toxicology experiments [4], each binary factor represents the presence or absence of a particular toxin. Scientists consider the absence of all toxins to be the natural reference for the possible presence of some toxins. In such experiments, the status of the absence of a toxin can be naturally regarded as a baseline level. Another practical example is introduced by Glonek and Solomon [5] in a leukemic mice experiment. The authors presented two binary components that stand for sample and time, respectively. A natural baseline level is the condition of the non-leukemic line for one factor and time zero for another factor. For a broader interpretation of BP, one is referred to [6,7].

In recent years, choosing efficient designs under BP has raised considerable attention from researchers. The *D*-optimality and *A*-optimality are two commonly used efficiency criteria for selecting optimal experimental designs under the main effect model. From an information perspective, they aim to maximize the information content of the experimental data or minimize the variance of parameter estimates. Mukerjee and Tang [7] highlighted the *A*-optimality of two-level orthogonal arrays for BP when the interaction effects are all absent. Mukerjee and Huda [8] applied approximate theory together with discretization procedures to find designs that have high *A*-efficiencies and are robust to model misspecification. Liu et al. [9] employed the *D*-optimality criterion to find designs under BP that are both efficient and robust.

For situations where the interaction effects are present, the experimenter usually concerns the bias caused by interaction effects in estimating the main effects. Karunanayaka and Tang [10] proposed to add runs to one-factor-at-a-time designs for generating compromise designs that are competitive under both the efficiency criterion and the bias criterion. Mukerjee and Tang [7] proposed an optimality criterion, the *K*-aberration criterion, which quantifies the bias for two-level designs. Under the *K*-aberration criterion, a few works on designs for BP emerged. Li et al. [11] proposed an efficient incomplete search algorithm for finding nearly optimal designs and tabulated some 20-run (nearly) optimal two-level designs. Miller and Tang [12] focused on identifying efficient two-level regular designs through bridging the *K*-values and word length pattern. Mukerjee and Tang [13] developed certain rank conditions which, in conjunction with the idea of the minimum moment aberration and recursive set, help alleviate the burden of finding optimal two-level regular designs. Lin and Yang [14] considered finding multistratum designs for BP by using the coordinate-exchange algorithm. Li et al. [15] further proposed a theoretical construction method of compromise designs. Chen et al. [16] considered the situations where some two-factor interactions are also of interest in addition to the main effects and proposed an algorithm for searching optimal designs under their proposed minimum aberration criterion. Sun and Tang [17] investigated the linear relationship between the effects under BP and those under OP and explored its applications to design construction under BP in terms of estimability, optimality, and robustness. Yan and Zhao [18,19] put forward a minimum aberration criterion for three-level designs for BP and found some optimal designs using their proposed construction algorithm.

This paper focuses on two-level regular designs under the *K*-aberration. Though the two-level nonregular designs may outperform the regular ones in some cases, there is a compelling reason for considering two-level regular designs as has been justified in [13]: the results on two-level regular designs serve as a benchmark for evaluating further work on nonregular ones that have to be compared. Given the importance of the two-level regular designs for BP, we endeavor to make further progress on bridging the *K*-values and word length pattern and analytically calculate the quantities for two-level regular designs with a higher resolution based on the results in [13]. Such new progress has the following advantages: (i) it can help screen out the candidate designs that are not likely to be *K*-aberration optimal and thus can yield a further simplification of the search algorithm in [13]; (ii) it is capable of finding two-level regular designs that have better or even optimal *K*-aberration characteristics than those identified by the results in [13]. Illustrative examples are given to demonstrate these points.

The rest of this paper is organized as follows. Section 2 introduces some necessary notation and elementary knowledge of BP and the word length pattern. Section 3 presents the main results of this paper. Applications of the theoretical results are included in Section 4. The concluding remarks are given in Section 5.

## 2. Preliminaries

Consider an experiment with *n* factors each at two levels. A full design includes $2^{n}$ runs that correspond to $2^{n}$ level combinations of the *n* factors. Suppose only a $2^{-m}$ fraction of the full design can be carried out for economic reasons. This paper considers the regular fraction of the full design. Let $2^{n-m}$ denote a two-level regular fractional factorial design *D* with $N=2^{n-m}$ runs and *n* columns, with each column at two levels 0 and 1; such a design can be obtained as follows: Let q=n−m. Define the N×(N−1) matrix

(1) $$H_{q}=\left(1,2,12,3,13,\ldots ,q,1q,\ldots ,12\ldots q\right),$$

with columns arranged in Yates order, where

$$\begin{array}{c} 1={\left(0,1,0,1,\ldots ,0,1,0,1\right)}_{2^{q}}^{\prime },\\ 2={\left(0,0,1,1,\ldots ,0,0,1,1\right)}_{2^{q}}^{\prime },\\ \ldots \\ q={\left(0,\ldots ,0,1,\ldots ,1\right)}_{2^{q}}^{\prime } \end{array}$$

are *q* independent columns, and the other columns are obtained by taking the component-wise sum (modulo 2) of the independent ones, and say $12={\left(0,1,1,0,\ldots ,0,1,1,0\right)}_{2^{q}}^{\prime }$. A regular $2^{n-m}$ design *D* can be obtained by selecting *n* columns of $H_{q}$ such that *q* are independent and the other m(=n−q) columns are component-wise sums (modulo 2) of the *q* independent ones. Clearly, the design *D* is an N×n submatrix of $H_{q}$.

For a regular $2^{n-m}$ design *D*, we regard it as a set with elements being the *n* columns of *D* and denote still the set as *D* without causing confusion. Let $\omega_{s}=\left\{g_{1},\ldots ,g_{s}\right\}$ denote a subset of *s* columns of *D* and $\Omega^{s}\left(D\right)$ denote the collection of all the possible $\omega_{s}$, where s≤n. For ease of presentation, $\Omega^{s}$ is sometimes used instead of $\Omega^{s}\left(D\right)$ without causing confusion. Then for any $\omega \in \Omega^{s}\left(D\right)$, ω is also an N×s submatrix of *D*. Note that each row of ω is an *s*-tuple with entries of 0 or 1; we call it a binary *s*-tuple. Let α(ω) be the number of times that the *s*-tuple 11⋯1 occurs in the submatrix ω. For a regular $2^{n-m}$ design *D*, let $K_{s}$ denote the total bias to the main effects estimations caused by all the *s*-factor interaction effects. Mukerjee and Tang [7] proved that

(2) $$K_{s}=\left(4/N^{2}\right)\left(sT_{1s}+T_{2s}\right),$$

where

(3) $$T_{1s}=\sum_{\omega_{s}\in \Omega^{s}\left(D\right)}{\left(\alpha \left(\omega_{s}\right)\right)}^{2},$$

$$T_{2s}=\sum_{\omega_{s+1}\in \Omega^{s+1}\left(D\right)}\sum_{\omega_{s}\in \Omega^{s}\left(\omega_{s+1}\right)}{\left(2\alpha \left(\omega_{s+1}\right)-\alpha \left(\omega_{s}\right)\right)}^{2},$$

and $\Omega^{s}\left(\omega_{s+1}\right)$ denotes the collection of all the possible $\omega_{s}\subset \omega_{s+1}$ for given $\omega_{s+1}\in \Omega^{s+1}\left(D\right)$. Formula (2) applies to any two-level designs not just the regular ones. For more details of the *K*-aberration, one is referred to [7]. A two-level orthogonal array is *K*-aberration optimal if it sequentially minimizes the following sequence:

(4) $$\left(K_{2},K_{3},\ldots ,K_{m}\right)$$

among all the two-level designs, given the run size and the number of columns.

The word length pattern (WLP) is a concept proposed for the designs under OP. With a slight modification, such a concept can also be applied to the two-level regular designs under BP as follows: For the original definition of WLP under OP, one is referred to [20]. For any regular $2^{n-m}$ baseline design *D*, denote $\psi (\omega_{k})$ as the sum of the components of the vector $g_{1}+\cdots +g_{k}$ (modulo 2), where $\omega_{k}=\left\{g_{1},\cdots ,g_{k}\right\}\in \Omega^{k}\left(D\right)$. Define

$$J_{k}\left(\omega_{k}\right)=\left|2\psi \left(\omega_{k}\right)-N\right|.$$

If the *k* columns in $\omega_{k}$ satisfy $g_{1}+\cdots +g_{k}$ (modulo 2) $=1_{N}$ or $0_{N}$, then we have $\psi (\omega_{k})=N$ or 0, which leads to $J_{k}\left(\omega_{k}\right)=N$ and the *k* columns correspond to a defining word, where $1_{N}$ and $0_{N}$ denote the *N*-vectors with all components being 1 or 0, respectively. If the *k* columns in $\omega_{k}$ do not correspond to a defining word, then these columns must be independent of each other. This means that all of the binary *k*-tuples appear equally often as rows in $\omega_{k}$, which leads to $J_{k}\left(\omega_{k}\right)=0$. Let $A_{k}\left(D\right)=\sum_{\omega_{k}\in \Omega^{k}\left(D\right)}J_{k}\left(\omega_{k}\right)/N$, then $A_{k}\left(D\right)$ is the number of defining words of length *k* of the regular design *D*. For a given two-level regular design, we call the corresponding sequence

(5) $$(A_{3},A_{4},A_{5},\ldots ,A_{n}),$$

as its word length pattern and *t* as its resolution, if the first nonzero element in Sequence (5) is $A_{t}$, where 3≤t≤n.

With the primary knowledge above, the next section builds connections between Sequences (4) and (5).

## 3. Main Results

Note that both Sequences (4) and (5) are closely related to the collection ω of a regular $2^{n-m}$ design. Recalling the definition of $T_{1s}$ in (3), when an *s*-column collection $\omega_{s}$ contains no defining word, all of the binary *s*-tuples appear exactly $N/2^{s}$ times in $\omega_{s}$, which means that such an $\omega_{s}$ contributes $N/2^{s}$ to $T_{1s}$. When an *s*-column collection $\omega_{s}$ contains some defining words, i.e., the columns in $\omega_{s}$ form some defining words, the analyses for the contribution to $T_{1s}$ caused by such an $\omega_{s}$ become complex. Similar analyses are also required when considering $T_{2s}$. Therefore, it is necessary to investigate the possible cases of a given collection $\omega_{s}$ containing defining words, so as to calculate $K_{s}$. Suppose a regular $2^{n-m}$ design has a resolution of *t*. Lemma 1 presents the maximum number of defining words in each of its (t+2)-column collections $\omega_{t+2}$.

**Lemma** **1.** *Suppose D is a regular $2^{n-m}$ design of resolution t and $\omega_{t+2}\in \Omega^{t+2}\left(D\right)$. Then*

(i) *$\omega_{t+2}$ contains at most two independent defining words for t=3,4;*
(ii) *$\omega_{t+2}$ contains at most one defining word for t≥5,*

*where t+2≤n.*

**Proof.**  (i) For t=3, denote $\omega_{5}=\left\{g_{1},g_{2},g_{3},g_{4},g_{5}\right\}$. If $\omega_{5}$ contains three independent defining words $W_{1}$, $W_{2}$, and $W_{3}$, then $W_{1}$, $W_{2}$, and $W_{3}$ generate another four defining words $W_{1}W_{2}$, $W_{1}W_{3}$, $W_{2}W_{3}$, and $W_{1}W_{2}W_{3}$. These seven defining words contain at least 21 letters (columns) since each defining word contains at least t(=3) letters. Note that a letter appears at most four times among the seven defining words. Then the seven defining words contain no more than 20 letters since there are only five columns in $\omega_{5}$. This contradiction shows the validity of (i) for t=3. For t=4, the proof is similar.

(ii) If $\omega_{t+2}$ contains two defining words $W_{1}$ and $W_{2}$ with the length $t_{1}$ and $t_{2}$, respectively, then $W_{1}$ and $W_{2}$ have at least $t_{1}+t_{2}-\left(t+2\right)$ common letters. Since $t_{1}\geq t$ and $t_{2}\geq t$, the length of the defining word $W_{1}W_{2}$ is at most $\left(t_{1}+t_{2}\right)-2\left[t_{1}+t_{2}-\left(t+2\right)\right]\leq 4$, which contradicts t≥5. This completes the proof of (ii).    □

Before proceeding to the main results of this section, we first introduce a lemma that is a refinement of the results from [12,21].

**Lemma** **2.** *Denote $g_{1},g_{2},\ldots ,g_{i},g_{i+1},\ldots ,g_{j}$ as j columns from a regular $2^{n-m}$ design D of resolution t. Suppose, among these j columns, only the first i ones correspond a defining word, i.e., $g_{1}+g_{2}+\cdots +g_{i}\left(modulo2\right)=0_{N}$ or $1_{N}$. Then, we have the following:*

(i) *The rows in the i-column matrix $\left(g_{1},g_{2},\ldots ,g_{i}\right)$ must consist of $N/2^{i-1}$ copies of a half replicate of the full $2^{i}$ factorial design;*
(ii) *Furthermore, in matrix $\left(g_{1},g_{2},\ldots ,g_{i},g_{i+1},\ldots ,g_{j}\right)$, for the $N/2^{i-1}$ copies of each distinct row of matrix $\left(g_{1},g_{2},\ldots ,g_{i}\right)$, all the $2^{j-i}$ distinct rows of matrix $\left(g_{i+1},\ldots ,g_{j}\right)$ appear equally $N/2^{j-1}$ times,*

*where 3≤t≤i<j≤n.*

In (i) of Lemma 2, the half replicate of the full $2^{i}$ factorial design that the *i*-column matrix $\left(g_{1},g_{2},\ldots ,g_{i}\right)$ contains depends on whether $g_{1}+g_{2}+\cdots +g_{i}=0_{N}$ or $1_{N}$ (modulo 2). This is addressed in detail in Remark 1.

**Remark** **1.** In Lemma 2, if $g_{1}+g_{2}+\cdots +g_{i}=0_{N}$ (modulo 2), then all the possible *i*-tuples that contain an even number of ones appear $N/2^{i-1}$ times in the *i*-column matrix $\left(g_{1},g_{2},\ldots ,g_{i}\right)$. If $g_{1}+g_{2}+\cdots +g_{i}=1_{N}$ (modulo 2), then all the possible *i*-tuples that contain an odd number of ones appear $N/2^{i-1}$ times in the *i*-column matrix $\left(g_{1},g_{2},\ldots ,g_{i}\right)$.

For a defining word *W*, let ϕ(W) denote the vector generated by taking component-wise sums (modulo 2) of the columns in the defining word. Then, $\phi =0_{N}$ or $1_{N}$. Denote $A_{i}^{0}$ and $A_{i}^{1}$ as the numbers of length *i* defining words with $\phi =0_{N}$ and $1_{N}$, respectively, where i=3,4,…,n. Clearly, $A_{i}^{0}+A_{i}^{1}=A_{i}.$ Denote $A_{4}^{0,0}$ as the number of pairs of length four defining words that have two common columns and $\phi =0_{N}$, and $A_{4}^{1,1}$ as the number of pairs of length four defining words that have two common columns and $\phi =1_{N}$. Theorem 1 builds the bridge between $K_{t+1}$ and the WLP for t=4.

**Theorem** **1.** *Suppose D is a regular $2^{n-m}$ design with resolution t=4, then*

(6) K5=(1/16)25n5+(n−4)(2n−15)A4+20(n−4)A40−4A40,0−8A41,1+(1/16)25(n−6)A5+20A51+6A6.

**Proof.**  We calculate $T_{15}$ and $T_{25}$, separately. For $T_{15}$, suppose $\omega_{5}=\left\{g_{1},g_{2},g_{3},g_{4},g_{5}\right\}\in \Omega^{5}\left(D\right)$. Since *D* has resolution 4, $\omega_{5}$ contains at most one defining word. There are five scenarios as follows:

(a1) $\omega_{5}$ contains one length-four defining word *W* with $\phi \left(W\right)=0_{N}$;
(a2) $\omega_{5}$ contains one length-four defining word *W* with $\phi \left(W\right)=1_{N}$;
(a3) $\omega_{5}$ contains one length-five defining word *W* with $\phi \left(W\right)=0_{N}$;
(a4) $\omega_{5}$ contains one length-five defining word *W* with $\phi \left(W\right)=1_{N}$;
(a5) The five columns in $\omega_{5}$ are independent of each other.

For (a1), suppose the defining word is $g_{1}+g_{2}+g_{3}+g_{4}=0_{N}$ (modulo 2) without loss of generality. According to Lemma 2 (i) and Remark 1, the rows that consist of four ones appear $N/2^{3}$ times in the four-column matrix $\left(g_{1},g_{2},g_{3},g_{4}\right)$. Therefore, the columns of entire ones appear $N/2^{4}$ times in the five-column matrix $\left(g_{1},g_{2},g_{3},g_{4},g_{5}\right)$, i.e., $\alpha (\omega_{5})=N/16$.

For (a2), suppose the defining word is $g_{1}+g_{2}+g_{3}+g_{4}=1_{N}$ (modulo 2) without loss of generality. According to Lemma 2 (i) and Remark 1, the four-column matrix $\left(g_{1},g_{2},g_{3},g_{4}\right)$ contains only rows that consist of an odd number of ones. This implies that none of the rows in matrix $\left(g_{1},g_{2},g_{3},g_{4},g_{5}\right)$ contains entire ones, i.e., $\alpha (\omega_{5})=0$.

With similar arguments to (a1) and (a2), we can obtain $\alpha (\omega_{5})=0$ and $\alpha (\omega_{5})=N/16$ for (a3) and (a4), respectively. It is obvious that $\alpha (\omega_{5})=N/32$ for (a5). The number of $\omega_{5}$s belonging to (a1)–(a5) are $\left(n-4\right)A_{4}^{0}$, $\left(n-4\right)A_{4}^{1}$, $A_{5}^{0}$, $A_{5}^{1}$, and n5−(n−4)A40−(n−4)A41−A50−A51, respectively. With the analysis above, it yields that

(7) T15=(N/16)2(n−4)A40+(N/16)2A51+(N/32)2n5−(n−4)A4−A5.

Now, we consider calculating $T_{25}$. According to Lemma 1 (i) for t=4, there are nine possibilities for $\omega_{6}\in \Omega^{6}\left(D\right)$:

(b1) $\omega_{6}$ contains two independent defining words $W_{1}$ and $W_{2}$, which generate the third defining word $W_{3}=W_{1}W_{2}$. Each of the three defining words has a length of four, one has $\phi =0_{N}$ and the other two have $\phi =1_{N}$;
(b2) $\omega_{6}$ contains two independent defining words $W_{1}$ and $W_{2}$, which generate the third defining word $W_{3}=W_{1}W_{2}$. Each of the three defining words has a length of four and $\phi =0_{N}$;
(b3) $\omega_{6}$ contains only one defining word with a length of four and $\phi =0_{N}$;
(b4) $\omega_{6}$ contains only one defining word with a length of four and $\phi =1_{N}$;
(b5) $\omega_{6}$ contains only one defining word with a length of five and $\phi =0_{N}$;
(b6) $\omega_{6}$ contains only one defining word with a length of five and $\phi =1_{N}$;
(b7) $\omega_{6}$ contains only one defining word with a length of six and $\phi =0_{N}$;
(b8) $\omega_{6}$ contains only one defining word with a length of six and $\phi =1_{N}$;
(b9) The six columns in $\omega_{6}$ are independent of each other.

For (b1)–(b9), denote $\omega_{5}$ as any five-column subset of $\omega_{6}$, i.e., $\omega_{5}\in \Omega^{5}\left(\omega_{6}\right)$. Now we proceed to investigate the values of $2\alpha \left(\omega_{6}\right)-\alpha \left(\omega_{5}\right)$ and the number of $\omega_{6}$s in each of the cases for (b1)–(b9).

For (b1), since there is a length-four defining word, say $W_{1}$, with $\phi \left(W_{1}\right)=1_{N}$ in $\omega_{6}$, none of the rows in the matrix consisting of the columns involved in $W_{1}$ contains entire ones and thus $\alpha (\omega_{6})=0$. With careful checking, each $\omega_{5}\in \Omega^{5}\left(\omega_{6}\right)$ must contain only one defining word, say *W*, which has a length of four with either $\phi \left(W\right)=0_{N}$ or $1_{N}$. For the $\omega_{5}$s of the former case, we have $\alpha \left(\omega_{5}\right)=N/2^{4}$ and there are two such $\omega_{5}$s in $\Omega^{5}\left(\omega_{6}\right)$. For the $\omega_{5}$s of the latter case, we have $\alpha (\omega_{5})=0$ and there are four such $\omega_{5}$s in $\Omega^{5}\left(\omega_{6}\right)$. Note that the $\omega_{6}$ in (b1) contains a pair of length-four defining words that have two columns in common and their $\phi =1_{N}$. Recalling the meaning of $A_{4}^{1,1}$, we conclude that the number of $\omega_{6}$s belonging to (b1) is $A_{4}^{1,1}$.

For (b2), since $\omega_{6}$ contains three length-four defining words and each of which has $\phi =0_{N}$, we have $\alpha \left(\omega_{6}\right)=N/2^{5}$ according to Lemma 2 (ii). Note that each $\omega_{5}\in \Omega^{5}\left(\omega_{6}\right)$ must contain one of these three defining words. From Lemma 2 (ii), we have $\alpha \left(\omega_{5}\right)=N/2^{4}$ for each $\omega_{5}\in \Omega^{5}\left(\omega_{6}\right)$ and there are six such $\omega_{5}$s in $\Omega^{5}\left(\omega_{6}\right)$. Note that there are three pairs of length-four defining words in $\omega_{6}$ and each pair has two columns in common. Thus, it has totally $A_{4}^{0,0}/3$ $\omega_{6}$s in (b2).

For (b3), it is easy to obtain that $\alpha \left(\omega_{6}\right)=N/2^{5}$. Each $\omega_{5}\in \Omega^{5}\left(\omega_{6}\right)$ contains either a length-four defining word with $\phi =0_{N}$ or five independent columns. For the $\omega_{5}$s of the former case, we have $\alpha \left(\omega_{5}\right)=N/2^{4}$ and there are two such $\omega_{5}$s in $\Omega^{5}\left(\omega_{6}\right)$. For the $\omega_{5}$s of the latter case, we have $\alpha \left(\omega_{5}\right)=N/2^{5}$ and there are four such $\omega_{5}$s in $\Omega^{5}\left(\omega_{6}\right)$. Now we investigate the number of $\omega_{6}$s that belong to (b3). The four columns of each of the $A_{4}^{0}$ defining words jointed with any two of the remaining n−4 columns of *D* induce an $\omega_{6}\in \Omega^{6}\left(D\right)$. Notably, the three defining words in each $\omega_{6}$ of case (b2) induce exactly the $\omega_{6}$ itself. Similarly, the $\omega_{6}$ in case (b1) can be induced by the length-four defining word with $\phi =0_{N}$ in it. Therefore, the number of $\omega_{6}$s in case (b3) is n−42A40−A40,0−2A41,1.

For (b4), we have $\alpha (\omega_{6})=0$ as there is a length-four defining word with $\phi =1_{N}$. With a similar analysis to (b3), among the six $\omega_{5}$s in $\Omega^{5}\left(\omega_{6}\right)$, two of them have $\alpha (\omega_{5})=0$ and four of them have $\alpha \left(\omega_{5}\right)=N/2^{5}$, depending on whether the $\omega_{5}$ contains a length-four defining word with $\phi =1_{N}$ or not. The four columns of each of the $A_{4}^{1}$ defining words jointed with any two of the remaining n−4 columns of *D* induce an $\omega_{6}$ in $\Omega^{6}\left(D\right)$. One thing to note is that the two length-four defining words with $\phi =1_{N}$ in each $\omega_{6}$ of case (b1) induce exactly the $\omega_{6}$ itself. The number of $\omega_{6}$s belonging to (b4) is n−42A41−2A41,1.

For cases (b5)–(b8), the results on the values of $\alpha (\omega_{6})$s, the number of $\omega_{6}$s belonging to each case, the values of $\alpha (\omega_{5})$s, and the number of $\omega_{5}$s in each $\Omega^{5}\left(\omega_{6}\right)$, which have the same values of $\alpha (\omega_{5})$s, are straightforward. These results are summarized in Table 1 along with those for cases (b1)–(b4), where the notation $n_{\omega_{5}}$ represents the number of $\alpha (\omega_{5})$s of each value. Note that each $\omega_{6}$ in (b9) has $\alpha \left(\omega_{6}\right)=N/2^{6}$ and $\alpha \left(\omega_{5}\right)=N/2^{5}$ for each $\omega_{5}\in \Omega^{5}\left(\omega_{6}\right)$; this results in $2\alpha \left(\omega_{6}\right)-\alpha \left(\omega_{5}\right)=0$. Therefore, there is no need to consider case (b9) when calculating $T_{25}$.

With Table 1, it is obtained that

(8) T25=(N/32)24n−42A4−4A40,0−8A41,1+5(n−5)A5+6A6.

With Equations (7) and (8), we obtain

K5=(4/N2)(5T15+T25)=(1/16)25n5+(n−4)(2n−15)A4+20(n−4)A40−4A40,0−8A41,1+(1/16)25(n−6)A5+20A51+6A6.

This completes the proof.    □

Theorem 2 below builds the relationship between $K_{t+1}$ and the WLP for t≥5.

**Theorem** **2.** *Suppose D is a regular $2^{n-m}$ design with resolution t≥5, then*

Kt+1=(1/22t)4(t+1)(n−t)At1+4(t+1)At+10+tn−t2−(t+1)(n−t)At+(1/22t)(t+1)(n−t−2)At+1+(t+2)At+2+(t+1)nt+1

*for an odd t≥5, and*

Kt+1=(1/22t)4(t+1)(n−t)At0+4(t+1)At+11+tn−t2−(t+1)(n−t)At+(1/22t)(t+1)(n−t−2)At+1+(t+2)At+2+(t+1)nt+1

*for an even t≥5.*

**Proof.**  To calculate $T_{1s}$ in (2) for s=t+1, consider five possibilities for $\omega_{t+1}\in \Omega^{t+1}\left(D\right)$:

(c1) $\omega_{t+1}$ contains only one defining word with length *t* and $\phi =0_{N}$;
(c2) $\omega_{t+1}$ contains only one defining word with length *t* and $\phi =1_{N}$;
(c3) $\omega_{t+1}$ contains only one defining word with length t+1 and $\phi =0_{N}$;
(c4) $\omega_{t+1}$ contains only one defining word with length t+1 and $\phi =1_{N}$;
(c5) $\omega_{t+1}$ consists of t+1 columns that are independent of each other.

With similar analyses to Theorem 1, we have Table 2 and Table 3 for calculating $T_{1(t+1)}$ for an odd and even t≥5, respectively. With Table 2 and Table 3, we obtain that

(9) T1(t+1)=(N/2t+1)24(n−t)At1+4At+10+nt+1−(n−t)At−At+1

for an odd t≥5, and

(10) T1(t+1)=(N/2t+1)24(n−t)At0+4At+11+nt+1−(n−t)At−At+1

for an even t≥5.

Considering $T_{2(t+1)}$, there are seven possibilities for $\omega_{t+2}\in \Omega^{t+2}\left(D\right)$:

(d1) $\omega_{t+2}$ contains only one defining word and its length is *t* with $\phi =0_{N}$;
(d2) $\omega_{t+2}$ contains only one defining word and its length is *t* with $\phi =1_{N}$;
(d3) $\omega_{t+2}$ contains only one defining word and its length is t+1 with $\phi =0_{N}$;
(d4) $\omega_{t+2}$ contains only one defining word and its length is t+1 with $\phi =1_{N}$;
(d5) $\omega_{t+2}$ contains only one defining word and its length is t+2 with $\phi =0_{N}$;
(d6) $\omega_{t+2}$ contains only one defining word and its length is t+2 with $\phi =1_{N}$;
(d7) $\omega_{t+2}$ consists of t+2 columns that are independent of each other.

With similar analyses to Theorem 1, we have Table 4 and Table 5 for calculating $T_{2(t+1)}$ for an odd and even t≥5, respectively. Note that each $\omega_{t+2}$ in (d7) has $\alpha \left(\omega_{t+2}\right)=N/2^{t+2}$ and $\alpha \left(\omega_{t+1}\right)=N/2^{t+1}$ for each $\omega_{t+1}\in \Omega^{t+1}\left(\omega_{t+2}\right)$; this results in $2\alpha \left(\omega_{t+2}\right)-\alpha \left(\omega_{t+1}\right)=0$. Therefore, there is no need to consider case (d7) when calculating $T_{2(t+1)}$.

With Table 4 and Table 5, we obtain that

(11) T2(t+1)=(N/2t+1)2tn−t2At+(t+1)(n−t−1)At+1+(t+2)At+2

for both an odd and even t≥5.

With Equations (9)–(11), we have

Kt+1=(4/N2)[(t+1)T1(t+1)+T2(t+1)]=(1/22t)4(t+1)(n−t)At1+4(t+1)At+10+tn−t2−(t+1)(n−t)At+(1/22t)(t+1)(n−t−2)At+1+(t+2)At+2+(t+1)nt+1

for an odd t≥5, and

Kt+1=(4/N2)[(t+1)T1(t+1)+T2(t+1)[=(1/22t)4(t+1)(n−t)At0+4(t+1)At+11+tn−t2−(t+1)(n−t)At+(1/22t)(t+1)(n−t−2)At+1+(t+2)At+2+(t+1)nt+1

for an even t≥5. This completes the proof.    □

**Remark** **2.** Theorems 1 and 2 establish relationships between the *K*-aberration and WLP that are further developments based on the work in [12]. Theorems 1 and 2 help narrow down the choice of finding optimal regular $2^{n-m}$ designs. Moreover, for some situations, Theorems 1 and 2 are capable of identifying the optimal ones. This point will be demonstrated in Section 4.

## 4. Applications

It is worth noting that the concept of isomorphism for the designs under BP is different from that under OP. Under OP, two designs are called isomorphic if one can be obtained from the other by column-permuting, row-permuting, or symbol-switching. However, the symbols of the two-level designs are not interchangeable under BP. Hence, two designs are called isomorphic under BP if one can be obtained from the other by column-permuting or row-permuting. Hereafter, we use the terms OP regular $2^{n-m}$ designs versus BP regular $2^{n-m}$ designs as discriminations. Clearly, switching symbols of some columns of OP regular $2^{n-m}$ designs may result in nonisomorphic BP designs. In the following, we illustrate how to find BP regular $2^{n-m}$ designs that have desirable *K*-aberration characteristics by using the catalogs of nonisomorphic OP regular $2^{n-m}$ designs displayed in [22].

Consider finding desirable BP regular $2^{16-10}$ designs under *K*-aberration. By checking the catalogs of nonisomorphic OP designs displayed in [22], all the OP regular $2^{16-10}$ designs have a resolution of either t=3 or 4. According to Theorem 1 in [12], any OP regular $2^{16-10}$ design with a resolution of t=4 has a smaller $K_{2}$ than those with a resolution of t=3, noting that K2=2n2(1/24−2)=60 for t=4 and K2=(1/22t−4)(t−1)nt−1+tAt=60+3A3/4 for t=3. Among the OP regular $2^{16-10}$ designs of resolution t=4, the designs with the minimum $A_{4}$ have a smaller $K_{3}$ according to Theorem 1 (b) in [12]. According to [22], the unique OP regular $2^{16-10}$ design with the minimum $A_{4}$, denoted as $D_{0}$, is determined by the following ten independent defining words: $g_{1}g_{2}g_{3}g_{4}g_{5}g_{7}$, $g_{1}g_{2}g_{3}g_{6}g_{8}$, $g_{1}g_{4}g_{6}g_{9}$, $g_{1}g_{2}g_{5}g_{6}g_{10}$, $g_{1}g_{3}g_{4}g_{11}$, $g_{1}g_{3}g_{5}g_{12}$, $g_{1}g_{2}g_{4}g_{13}$, $g_{3}g_{5}g_{6}g_{14}$, $g_{2}g_{4}g_{5}g_{6}g_{15}$, and $g_{2}g_{3}g_{5}g_{16}$, where $g_{1},\ldots ,g_{6}$ are the 1st, 2nd, $2^{2}$th, …,$2^{5}$th columns of the matrix $H_{q}$ in (1) with q=6. The ϕs of these ten defining words equaling to $0_{N}$ or $1_{N}$ determines $2^{10}$ BP regular designs that may have different *K*-aberration performances. According to Theorem 2 of [12], among the $2^{10}$ BP designs, those with the minimum $A_{4}^{0}$ have a smaller $K_{4}$. For example, the following two BP regular $2^{16-10}$ designs $D_{1}$ and $D_{2}$ have the minimum $A_{4}^{0}=17$, which results in the minimum $K_{4}=153.8906$:

$$\begin{array}{cccc} D_{1}: & \phi \left(g_{1}g_{2}g_{3}g_{4}g_{5}g_{7}\right)=1_{N}, & \phi \left(g_{1}g_{2}g_{3}g_{6}g_{8}\right)=0_{N}, & \phi \left(g_{1}g_{4}g_{6}g_{9}\right)=1_{N},\\ & \phi \left(g_{1}g_{2}g_{5}g_{6}g_{10}\right)=1_{N}, & \phi \left(g_{1}g_{3}g_{4}g_{11}\right)=1_{N}, & \phi \left(g_{1}g_{3}g_{5}g_{12}\right)=0_{N},\\ & \phi \left(g_{1}g_{2}g_{4}g_{13}\right)=0_{N}, & \phi \left(g_{3}g_{5}g_{6}g_{14}\right)=1_{N}, & \phi \left(g_{2}g_{4}g_{5}g_{6}g_{15}\right)=0_{N},\\ & \phi \left(g_{2}g_{3}g_{5}g_{16}\right)=1_{N}. & & \\ D_{2}: & \phi \left(g_{1}g_{2}g_{3}g_{4}g_{5}g_{7}\right)=1_{N}, & \phi \left(g_{1}g_{2}g_{3}g_{6}g_{8}\right)=0_{N}, & \phi \left(g_{1}g_{4}g_{6}g_{9}\right)=1_{N},\\ & \phi \left(g_{1}g_{2}g_{5}g_{6}g_{10}\right)=1_{N}, & \phi \left(g_{1}g_{3}g_{4}g_{11}\right)=1_{N}, & \phi \left(g_{1}g_{3}g_{5}g_{12}\right)=0_{N},\\ & \phi \left(g_{1}g_{2}g_{4}g_{13}\right)=0_{N}, & \phi \left(g_{3}g_{5}g_{6}g_{14}\right)=0_{N}, & \phi \left(g_{2}g_{4}g_{5}g_{6}g_{15}\right)=0_{N},\\ & \phi \left(g_{2}g_{3}g_{5}g_{16}\right)=1_{N}. & \end{array}$$

Although $D_{1}$ and $D_{2}$ have the same value of $K_{4}$, they can be discriminated with respect to $K_{5}$ by applying Theorem 1. Compared to $D_{1}$, $D_{2}$ has the same $A_{4}^{0,0}$ and $A_{4}^{1,1}$ but a smaller $A_{5}^{1}$ than $D_{1}$. This means that $D_{2}$ has a smaller $K_{5}$ than $D_{1}$ according to Theorem 1. As a confirmation, we calculate the $K_{s}$ values of the previously stated $2^{10}$ BP regular $2^{16-10}$ designs, and it transpires that $D_{2}$ is one of the *K*-aberration optimal BP regular $2^{10-6}$ designs.

Here is an example of the application of Theorem 2. Consider finding BP regular $2^{10-3}$ designs that have desirable *K*-aberration characteristics. By checking the catalogs of nonisomorphic OP regular $2^{10-3}$ designs provided in [22], we only need to consider the OP regular $2^{10-3}$ design, denoted as $D_{3}$, determined by the independent defining words $g_{1}g_{2}g_{3}g_{4}g_{5}g_{8}$, $g_{1}g_{2}g_{4}g_{6}g_{9}$, and $g_{1}g_{2}g_{3}g_{6}g_{7}g_{10}$, since it has $A_{4}=0$ and the minimum $A_{5}$ among all the nonisomorphic regular $2^{10-3}$ designs, where $g_{i}$ is the $2^{i-1}$th column of the matrix $H_{q}$ in (1) with q=7, i=1,…,7. There are $2^{3}$ BP regular $2^{10-3}$ designs associated with $D_{3}$ depending on whether the previously mentioned three defining words are equal to $0_{N}$ or $1_{N}$. Among these regular BP $2^{10-3}$ designs, those with the minimum $A_{5}^{0}$ have the minimum $K_{5}$ according to Theorem 2 of [12]. For example, the regular BP $2^{10-3}$ design, denoted as $D_{4}$, which is determined by the defining words $g_{1}g_{2}g_{3}g_{4}g_{5}g_{8}=0_{N}$, $g_{1}g_{2}g_{4}g_{6}g_{9}=0_{N}$, and $g_{1}g_{2}g_{3}g_{6}g_{7}g_{10}=0_{N}$, has the minimum $A_{5}^{0}=3$ and then the minimum $K_{5}=5.227$. At the same time, $D_{4}$ has the minimum $A_{6}^{0}=3$, which indicates that $D_{4}$ has the minimum $K_{6}$ according to Theorem 2. As a confirmation, we calculate the $K_{s}$ values of all the $2^{3}$ BP regular designs and find that $D_{4}$ is one of the *K*-aberration optimal BP regular $2^{10-3}$ designs.

The two examples above show that Theorems 1 and 2 can help to filter or select designs. Take Theorem 1 as an example. When the experimenters need to compare $2^{n-m}$ designs with resolution 4, they can firstly calculate $A_{4}$, $A_{4}^{0}$, $A_{4}^{0,0}$, $A_{4}^{1,1}$, $A_{5}$, $A_{5}^{1}$, and $A_{6}$ according to the defining words of the designs and then $K_{5}$ according to Theorem 1. Clearly, this is time-saving compared with calculating $K_{5}$ among the $2^{10}$ designs.

The results of Theorems 1 and 2 establish a relation between the *K*-values and word length pattern of a design. To facilitate practitioners in other fields applying the methods, the following algorithm is provided based on Theorem 1. The algorithm (Algorithm 1) can be directly extended if Theorem 2 is required.

**Algorithm 1:** For a given *n* and *m*, consider a $2^{n-m}$ design *D* with resolution 4.
Step 1.List all the defining words of *D*.Step 2.Calculate $A_{4}$, $A_{4}^{0}$, $A_{4}^{0,0}$, $A_{4}^{1,1}$, $A_{5}$, $A_{5}^{1}$, and $A_{6}$ according to the defining words.Step 3.Calculate $K_{5}$ using (6) directively.


Here, we would like to point out that, to calculate the defining words of *D*, one should refer to [20].

## 5. Concluding Remarks

In experiments with each factor having a null state or baseline level, the BP model has quite a natural explanation. Then, finding the optimal fractional factorial designs under BP becomes important. However, the number of nonisomorphic designs under BP is much larger than that under OP, which makes it intricate for us in finding the optimal designs. Together with the results in [12], the present work helps narrow down the choice of finding the optimal regular $2^{n-m}$ designs through bridging the *K*-values and WLP. Nonregular designs are commonly used in various experiments due to their flexibility of the run size. Therefore, further study that focuses on finding the optimal nonregular designs under BP is deserved. However, just like the regular designs, it is also intricate in finding the optimal nonregular designs under BP. An algorithm reducing the candidates of the optimal designs would be very useful.

## Figures and Tables

**Table 1:** $\alpha (\omega_{6})$ and $\alpha (\omega_{5})$ for calculating $T_{25}$ in Theorem 1.

Scenario	$\alpha (\omega_{6})$	$\alpha \left(\omega_{5}\right):n_{\omega_{5}}$	$n_{\omega_{6}}$
(b1)	0	N/16:2 and 0:4	$A_{4}^{1,1}$
(b2)	N/32	N/16:6	$A_{4}^{0,0}/3$
(b3)	N/32	N/16:2 and N/32:4	n−42A40−A40,0−2A41,1
(b4)	0	0:2 and N/32:4	n−42A41−2A41,1
(b5)	0	N/32:5 and 0:1	$\left(n-5\right)A_{5}^{0}$
(b6)	N/32	N/32:5 and N/16:1	$\left(n-5\right)A_{5}^{1}$
(b7)	N/32	N/32:6	$A_{6}^{0}$
(b8)	0	N/32:6	$A_{6}^{1}$

$n_{\omega_{5}}$: the number of $\alpha (\omega_{5})$s of each value for $\omega_{6}$. $n_{\omega_{6}}$: the number of $\omega_{6}$s in (b1)–(b8).

**Table 2:** $\alpha (\omega_{t+1})$ for calculating $T_{1(t+1)}$ for an odd t≥5 in Theorem 2.

	$\alpha (\omega_{t+1})$	$n_{\omega_{t+1}}$
(c1)	0	$\left(n-t\right)A_{t}^{0}$
(c2)	$N/2^{t}$	$\left(n-t\right)A_{t}^{1}$
(c3)	$N/2^{t}$	$A_{t+1}^{0}$
(c4)	0	$A_{t+1}^{1}$
(c5)	$N/2^{t+1}$	nt+1−(n−t)At−At+1

$n_{\omega_{t+1}}$: the number of $\omega_{t+1}$s in (c1)–(c5).

**Table 3:** $\alpha (\omega_{t+1})$ for calculating $T_{1(t+1)}$ for an even t≥5 in Theorem 2.

	$\alpha (\omega_{t+1})$	$n_{\omega_{t+1}}$
(c1)	$N/2^{t}$	$\left(n-t\right)A_{t}^{0}$
(c2)	0	$\left(n-t\right)A_{t}^{1}$
(c3)	0	$A_{t+1}^{0}$
(c4)	$N/2^{t}$	$A_{t+1}^{1}$
(c5)	$N/2^{t+1}$	nt+1−(n−t)At−At+1

$n_{\omega_{t+1}}$: the number of $\omega_{t+1}$s in (c1)–(c5).

**Table 4:** $\alpha (\omega_{t+2})$ and $\alpha (\omega_{t+1})$ for calculating $T_{2(t+1)}$ for an odd t≥5 in Theorem 2.

	$\alpha (\omega_{t+2})$	$\alpha \left(\omega_{t+1}\right):n_{\omega_{t+1}}$	$n_{\omega_{t+2}}$
(d1)	0	$N/2^{t+1}:t\;\mathrm{or}\;0:2$	n−t2At0
(d2)	$N/2^{t+1}$	$N/2^{t+1}:t\;\mathrm{or}\;N/2^{t}:2$	n−t2At1
(d3)	$N/2^{t+1}$	$N/2^{t+1}:\left(t+1\right)\;\mathrm{or}\;N/2^{t}:1$	$\left(n-t-1\right)A_{t+1}^{0}$
(d4)	0	$N/2^{t+1}:\left(t+1\right)\;\mathrm{or}\;0:1$	$\left(n-t-1\right)A_{t+1}^{1}$
(d5)	0	$N/2^{t+1}:\left(t+2\right)$	$A_{t+2}^{0}$
(d6)	$N/2^{t+1}$	$N/2^{t+1}:\left(t+2\right)$	$A_{t+2}^{1}$

$n_{\omega_{t+1}}$: the number of $\alpha (\omega_{t+1})$s of each value for $\omega_{t+2}$. $n_{\omega_{t+2}}$: the number of $\omega_{t+2}$s in (d1)–(d6).

**Table 5:** $\alpha (\omega_{t+2})$ and $\alpha (\omega_{t+1})$ for calculating $T_{2(t+1)}$ for an even t≥5 in Theorem 2.

	$\alpha (\omega_{t+2})$	$\alpha \left(\omega_{t+1}\right):n_{\omega_{t+1}}$	$n_{\omega_{t+2}}$
(d1)	$N/2^{t+1}$	$N/2^{t}:2\;\mathrm{or}\;N/2^{t+1}:t$	n−t2At0
(d2)	0	$N/2^{t+1}:t\;\mathrm{or}\;0:2$	n−t2At1
(d3)	0	$N/2^{t+1}:\left(t+1\right)\;\mathrm{or}\;0:1$	$\left(n-t-1\right)A_{t+1}^{0}$
(d4)	$N/2^{t+1}$	$N/2^{t+1}:\left(t+1\right)\;\mathrm{or}\;N/2^{t}:1$	$\left(n-t-1\right)A_{t+1}^{1}$
(d5)	$N/2^{t+1}$	$N/2^{t+1}:\left(t+2\right)$	$A_{t+2}^{0}$
(d6)	0	$N/2^{t+1}:\left(t+2\right)$	$A_{t+2}^{1}$

$n_{\omega_{t+1}}$: the number of $\alpha (\omega_{t+1})$s of each value for $\omega_{t+2}$. $n_{\omega_{t+2}}$: the number of $\omega_{t+2}$s in (d1)–(d6).

## Data Availability

No new data were created or analyzed in this study. Data sharing is not applicable to this article.
